# Spatial non-equilibrium and distribution dynamic evolution of the development level of national physical fitness in China’s provinces

**DOI:** 10.1371/journal.pone.0287806

**Published:** 2024-08-07

**Authors:** Qin Xiao, Haiting Xiao

**Affiliations:** 1 School of Physical Education, Hunan First Normal University, Changsha, Hunan, China; 2 School of Physical Education, Huizhou University, Huizhou, Guangdong, China; East China Normal University, CHINA

## Abstract

**Introduction:**

Physical health is fundamental to a country’s socio-economic advancement. An in-depth exploration of the spatial distribution and dynamic evolution of national physical fitness across China is crucial for enhancing the country’s overall physical health. This study aims to provide valuable insights into the geographical and temporal patterns of physical fitness, informing strategies for national physical fitness improvement.

**Methods:**

Employing data from China’s national physical fitness monitoring of 31 provinces, cities, and districts for the years 2005, 2010, and 2015, this study utilizes the Gini coefficient, its decomposition, and nonparametric density estimation methods. These techniques are applied to analyze the spatial disparities and temporal trends in national physical fitness levels among different demographics, including the overall population, males, females, and urban and rural residents.

**Results:**

The study reveals that the regional disparity in China’s national physical fitness initially narrowed and then expanded across the general population, females, and in both urban and rural areas, while consistently increasing among males. In terms of geographical distribution, the East, Middle, and West regions show significant heterogeneity, with the East-West gap being the most pronounced (Gini coefficients of 0.0249, 0.0230, 0.0263). The contribution rate of regional gaps was highest (54.40% -64.69%), followed by regional disparities (24.78% -27.15%), and the contribution of hypervariable density difference was the smallest (10.53% -19.75%). Despite a slight improvement in overall national physical fitness, the absolute regional disparities have further widened.

**Conclusions:**

Provinces with lower levels of national physical fitness demonstrate a ’club convergence’ trend, indicating regional clustering of similar fitness levels. Additionally, a ’catch-up effect’ is evident in rural areas, particularly in provinces with historically lower levels of national physical fitness. These findings suggest the need for region-specific public health strategies to address the growing disparities in national physical fitness across China.

## Introduction

With the acceleration of globalization and the increase in population flow, differences in health status and physical fitness levels across various countries and regions have emerged as critical issues in global public health. National physical fitness, as an integral component of physical fitness, reflects the comprehensive strength, competitiveness, and social progression of a country [1]. In China, the physical fitness of its population has become a focal point, especially in the context of striving towards a comprehensive well-off society. The "Healthy China 2030" plan, issued by the CPC Central Committee and the State Council, emphasizes enhancing the health quality of the Chinese nation and aligning it with the country’s socio-economic development [2]. Therefore, investigating the spatial characteristics and dynamic evolution of China’s national physical fitness development level is of considerable theoretical and practical importance. This research not only aids in promoting the "Healthy China 2030" initiative but also provides valuable insights for other developing countries aiming to enhance physical health.

To systematically understand the foundational aspects and evolving trends of national physical fitness, the General Administration of Sport of China has initiated a bi-decade physical fitness monitoring program, commencing from 2000. This program employs a random cluster sampling methodology and encompasses a broad demographic spectrum of Chinese citizens aged 3 to 69. The assessment comprises 24 distinct indicators, categorized under four primary dimensions: body composition, physiological functions, physical fitness, and health status.

The National Physical Fitness Composite Index serves as a pivotal metric in this initiative, synthesizing these indicators through a dimensionless analytical process. This composite index primarily offers a holistic view of the populace’s fitness status, effectively aggregating individual data points. Originating from the inaugural 2000 national physical fitness survey, the index established a baseline value of 100. Fluctuations in subsequent years pivot around this benchmark, with higher index values signifying superior levels of national physical fitness progression.

Hence, the focus of this study encompasses the comprehensive data spanning five distinct categories of the National Physical Fitness Composite Indexes across China’s 31 provinces. This dataset, drawn from the 2005, 2010, and 2015 national physical fitness surveys, includes comprehensive insights from the overall population, urban and rural residents, and gender-specific analysis for both males and females.

International studies on national physical fitness have primarily focused on two key aspects: the factors influencing physical fitness and the monitoring systems. Research has delved into economic, physical activity, and environmental factors affecting physical fitness, uncovering notable disparities among different socio-economic and geographical groups [3–6]. Furthermore, studies on monitoring systems have evaluated policies related to nutrition and physical activity in addressing health issues like obesity [7–9].

In China, recent research in sports and health has increasingly centered on national physical fitness, examining both the development levels and influencing factors among specific groups and the regional distribution of national physical fitness [10–17]. However, there is a lack of comprehensive exploration regarding the spatio-temporal heterogeneity of national physical fitness. Most domestic research has been limited to younger populations, with less emphasis on the physical development of middle-aged and elderly groups. Additionally, comparative studies on urban-rural and gender-based disparities in national physical fitness have been scarce.

This paper seeks to fill these gaps by employing the Gini coefficient and its sub-sample decomposition method, alongside nonparametric density estimation, to explore the spatial distribution and dynamic evolution of China’s national physical fitness. The study aims to provide a comprehensive understanding of regional disparities and trends in national physical fitness across different dimensions and areas. It also examines the impact of these disparities on the spatial imbalance of national physical fitness and explores the diverse patterns of dynamic evolution in different regions. This comprehensive approach will offer insights into the development of national physical fitness, contributing to policy-making for health improvement in China and beyond.

## Methods

### Gini coefficient and its decomposition method

Based on the national physical fitness monitoring data of 31 provinces (cities, districts) in China in 2005, 2010, and 2015, the Gini coefficient and its decomposition method and nonparametric density estimation method were used to study the population, males, females, urban and rural nationals in China. The data underlying this article were tested by specialized testers organized by the General Administration of Sports of China, all with the consent of the students and their guardians, and the data are publicly available and free of charge, these data were approved for use by the Biomedical Research Ethics Review Committee of Hunan Normal University (2022/388).

As one of the classic methods for the study of differences in various fields, the Gini coefficient and its decomposition method can overcome the problems that the Theil index and the traditional Gini coefficient cannot effectively solve the problems of overlapping and overlapping sample data and cannot further analyze the composition of regional differences and their sources (Dagum, 2008). 1997). In this paper, the Gini coefficient and its decomposition method are used to analyze the spatial disequilibrium degree, composition, and origin of the development level of Chinese national physical fitness. Its specific definition is shown in Formula (1):

$$G=\frac{\sum_{j=1}^{k}\sum_{h=1}^{k}\sum_{i=1}^{n_{j}}\sum_{r=1}^{n_{h}}\left|y_{ji}-y_{hr}\right|}{2\;\bar{y}n^{2}}$$ (1)

Among them, $\bar{y}$ represents the overall level of the development of national physical fitness in China, and the value is expressed by the average value of the development level of national physical fitness in all provinces (cities and autonomous regions) in the country; *y*_*ji*_ (*y*_*hr*_) represents the development level of national physical fitness in any province in the *j*(*h*) region, *n* represents the total number of provinces, *k* represents the number of divided regions, and *n*_*j*_ (*n*_*h*_) represents the number of provinces in the *j*(*h*) region. In order to further analyze the composition and source of the spatial disequilibrium in the development level of national physical fitness in the province, it is first necessary to rank the average development level of national physical fitness in the region, as shown in Formula (2):

$$\bar{Y_{1}}\leq \bar{Y_{h}}\leq \cdots \bar{Y_{j}}\leq \cdots \bar{Y_{k}}$$ (2)

According to the decomposition method of Dagum (1997), G can be further decomposed into the sum of the contribution of intra-regional gaps (*G*_*w*_), the contribution of inter-regional gaps (*G*_*nb*_), and the contribution of hypervariable density (*G*_*t*_), namely:

$$G=G_{w}+G_{nb}+G_{t}$$ (3)

The definitions of *G*_*w*_、*G*_*nb*_、*G*_*t*_, are shown in Eqs (4)–(6) respectively:

$$G_{w}=\sum_{j=1}^{k}G_{jj}p_{j}s_{j}$$ (4)

$$G_{nb}=\sum_{j=2}^{k}\sum_{h=1}^{j-1}G_{jh}(p_{j}s_{h}+p_{h}s_{j})D_{jh}$$ (5)

$$G_{t}=\sum_{j=2}^{k}\sum_{h=1}^{j-1}G_{jh}(p_{j}s_{h}+p_{h}s_{j})(1-D_{jh})$$ (6)

Among them, $p_{j}=\frac{n_{j}}{n}$, $s_{j}=\frac{n_{j}\bar{Y_{j}}}{n\bar{Y}}$, *j* = 1,2,3,⋯,*k*。 *G*_*jj*_ represents the Gini coefficient of the development level of national physical fitness in the *j* region, *G*_*jh*_ represents the Gini coefficient of the development level of national physical fitness between *j*、*h* regions, *D*_*jh*_ represents the degree of influence between regions relative to the contribution rate of the national physical fitness development *j*(*h*) level, the definitions are shown in Eqs (7)–(9)。

$$G_{jj}=\frac{\sum_{i=1}^{n_{j}}\sum_{r=1}^{n_{h}}\left|y_{ji}-y_{jr}\right|}{2\bar{Y_{j}}{n_{j}}^{2}}$$ (7)

$$G_{jh}=\frac{\sum_{i=1}^{n_{j}}\sum_{r=1}^{n_{h}}\left|y_{ji}-y_{jr}\right|}{n_{j}n_{h}(\bar{Y_{j}}+\bar{Y_{h}})}$$ (8)

$$D_{jh}=\frac{d_{jh}-p_{jh}}{d_{jh}+p_{jh}}$$ (9)

In the Formula 9, *d*_*jh*_ is the difference in the contribution rate of the development level of national physical fitness between regions, *p*_*jh*_ is the hypervariable first-order moment, which is defined as Formulas (10) and (11) respectively.


$$d_{jh}=\int_{0}^{\infty }dF_{j}(y)\int_{0}^{y}\left(y-x)dF_{h}(x\right)$$ (10)

$$p_{jh}=\int_{0}^{\infty }dF_{h}(y)\int_{0}^{y}\left(y-x)dF_{j}(x\right)$$ (11)

Among them, *F*_*j*_ (*F*_*h*_) represents cumulative density distribution function of area *j h*。

### The estimation models of nonparametric density *Kernel*

In this paper, kernel density estimation is used to further analyze the dynamic evolution characteristics of provincial national physical fitness development level. Density estimation is a non-parametric statistical method for fitting the density function of sample distribution in statistical problems feature.

For the dataset {*x*_1_,*x*_2_⋯*x*_*n*_}, the basic form of the density estimation *Kernel* function is

$$f_{h}(x)=\frac{1}{nh}\sum_{i=1}^{n}K|\frac{x_{i}-x}{h}|$$ (12)

Where is the h bandwidth, N is the number of observations, x is the average value of the observations, *k*(•) representing the kernel density, which is a weighting function or a smooth transition function, usually satisfying:

$$\left\{ \begin{array}{l} \underset{x\to \infty }{\lim}K(x)•x=0\\ K(x)\geq 0,\int_{-\infty }^{+\infty }K(x)dx=1\\ \sup K(x)<+\infty ,\int_{-\infty }^{+\infty }K^{2}(x)dx<+\infty \end{array}\right.$$ (13)

The *Kernel* function mainly includes four types: Gaussian kernel, exponential kernel, triangular kernel and quartic kernel. In this paper, Gaussian kernel is selected to estimate the dynamic evolution law of the development level of national physical fitness in China’s provinces. Its function form is:

$$f(x)=\frac{1}{\sqrt{2\pi }}\exp(-\frac{x^{2}}{2})$$ (14)

After the function is estimated, the dynamic evolution trend of the variable under investigation can be judged by examining the distribution position, shape and ductility of the variable kernel density estimation curve.

### Data sources and regional definition

This paper uses National Physical Fitness Composite Index in China’s provinces (cities and districts) to measure the development level of national physical fitness in each province. The data mainly includes three aspects of body shape, physical function and physical quality, and involves five categories of National Physical Fitness Composite Index of 31 provinces (cities, districts), towns, villages, males, and females, which are very representative. Since the survey did not cover regions such as Hong Kong, Macau, and Taiwan, these three regions were not included in the analysis. In order to ensure the comparability of the data, all the data are processed uniformly with the first national physical fitness monitoring data in 2000 as the base. Due to the large differences in economy, culture, infrastructure, etc. between regions, this paper further divides the 31 provinces into eastern (including 11 provinces) and central (including 8 provinces) according to the regional division standards of the National Bureau of Statistics), the west (including 12 provinces) three major regions.

## Results

### Visual description of spatial disequilibrium in the development level of national physical fitness in China’s provinces

**Fig 1:**
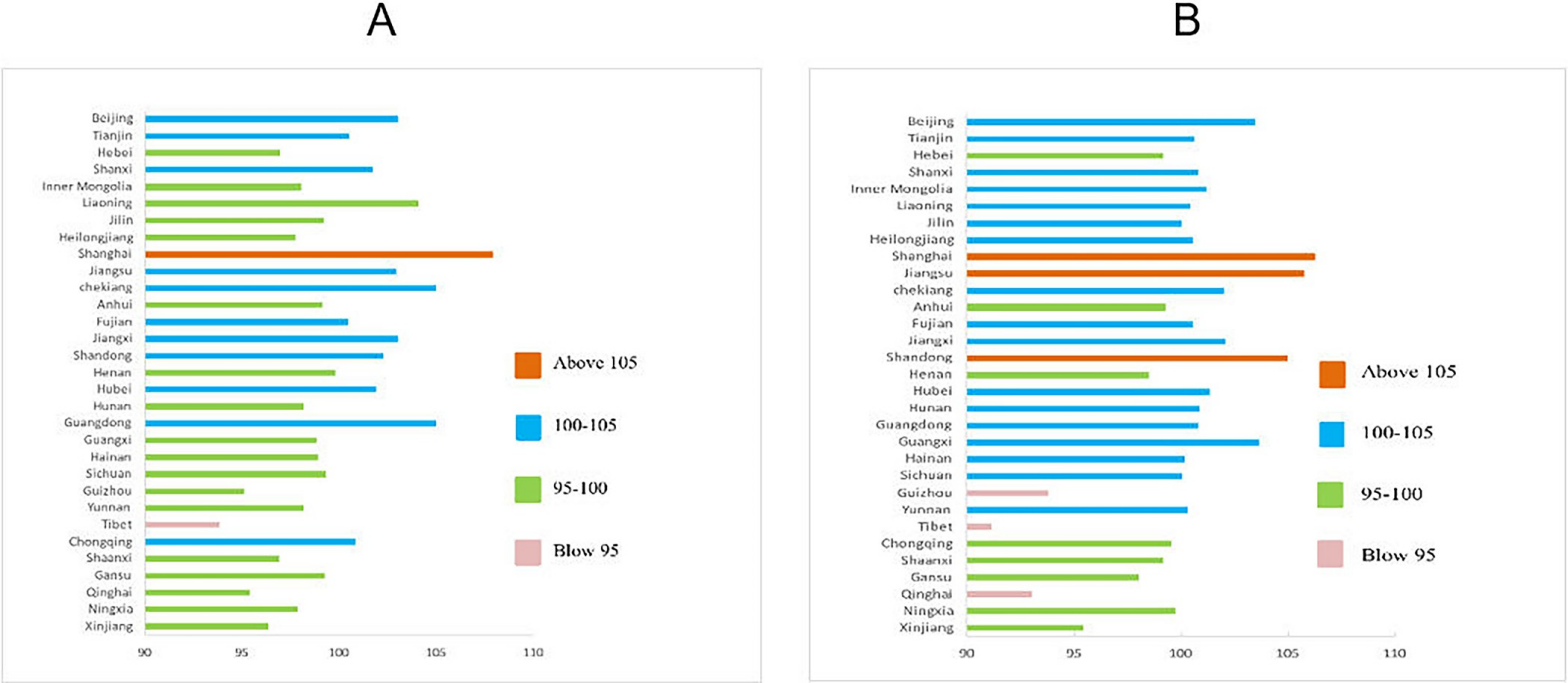
Bar chart of regional distribution of the overall level of Chinese national physical fitness (A for 2005 year, B for 2015 year).

With the help of Excel software, the Bar chart of the overall development level of Chinese national physical fitness in 2005 and 2015 was drawn, as shown in Fig 1. From this, it can be seen that there is a significant spatial disequilibrium in the development level of national physical fitness in China’s provinces. Compared with 2005, in 2015, the number of provinces with the National Physical Fitness Composite Index above 100 decreased significantly, and the number of provinces between 95 and 100 increased significantly. Although Shanghai was the province with the highest level of national physical fitness development in 2005 and 2015, its National Physical Fitness Composite Index dropped by 1.67 compared with 2005. Jiangsu ranked second in national physical fitness level in 2005 (the comprehensive index was 105.7), in 2015, the national physical fitness level dropped significantly (the comprehensive index was 102.92); and Zhejiang ranked second in the country with a comprehensive index of 104.99. In 2005 and 2015, the three provinces of Tibet, Qinghai and Guizhou ranked the bottom three in the country in terms of national physical fitness.

### The composition and source analysis of the spatial disequilibrium in the development level of Chinese national physical fitness

In order to further analyze the regional disparities in the spatial distribution of the development level of China’s national health and fitness, its composition, and sources, we used the Gini coefficient and its decomposition method to calculate the overall, male, female, urban, and rural national physical fitness in 2005, 2010 and 2015, respectively. The Gini coefficient of the physical development level is further decomposed according to the three major regions of the east, the middle, and the west. The results are shown in Tables 1–3.

**Table 1:** Regional Gini coefficients and their decomposition results of the development level of China’s overall national physical fitness.

years	Overall Coefficient	intra-regional Gini coefficient			Interregional Gini Coefficient			Contribution rate (%)		
		east	Central	west	East-Central	East-West	Central-West	within the area	Interregional	Hypervariable density
2005	0.0170	0.0127	0.0057	0.0205	0.0115	0.0249	0.0184	0.2715	0.6130	0.1155
2010	0.0167	0.0121	0.0094	0.0158	0.0176	0.0230	0.0143	0.2580	0.5444	0.1975
2015	0.0177	0.0162	0.0100	0.0118	0.0176	0.0263	0.0147	0.2478	0.6469	0.1053

**Table 2:** Regional Gini coefficients of male and female national physical fitness development levels and their decomposition.

Category	years	Overall coefficient	intra-regional Gini coefficient			Interregional Gini Coefficient			Contribution rate (%)		
			east	Central	west	East-Central	East-West	Central-West	within the area	Interregional	Hypervariable density
male	2005	0.0153	0.0104	0.0069	0.0182	0.0104	0.0221	0.0169	0.2717	0.6022	0.1261
	2010	0.0154	0.0125	0.0100	0.0136	0.0170	0.0203	0.0127	0.2681	0.5153	0.2167
	2015	0.0159	0.0157	0.0103	0.0105	0.0162	0.0225	0.0133	0.2628	0.5985	0.1387
female	2005	0.0195	0.0154	0.0069	0.0232	0.0138	0.0279	0.0207	0.2774	0.5816	0.1410
	2010	0.0185	0.0143	0.0102	0.0177	0.0186	0.0258	0.0160	0.2633	0.5508	0.1860
	2015	0.0199	0.0177	0.0105	0.0145	0.0196	0.0298	0.0167	0.2481	0.6516	0.1004

**Table 3:** Regional Gini coefficient and its decomposition of the development level of urban and rural national physical fitness.

category	years	Overall coefficient	intra-regional Gini coefficient			Interregional Gini Coefficient			Contribution rate (%)		
			east	Central	west	East-Central	East-West	Central-West	within the area	Interregional	Hypervariable density
town	2005	0.0189	0.0152	0.0075	0.0210	0.0145	0.0270	0.0193	0.2728	0.5826	0.1447
	2010	0.0182	0.0193	0.0090	0.0168	0.0196	0.0235	0.0143	0.2920	0.4486	0.2594
	2015	0.0238	0.0374	0.0109	0.0109	0.0271	0.0322	0.0149	0.2953	0.2416	0.4632
rural	2005	0.0182	0.0126	0.0097	0.0232	0.0119	0.0243	0.0212	0.2907	0.4935	0.2158
	2010	0.0163	0.0110	0.0097	0.0155	0.0153	0.0231	0.0155	0.2542	0.5705	0.1752
	2015	0.0181	0.0153	0.0099	0.0133	0.0175	0.0270	0.0158	0.2449	0.6436	0.1115

### Regional disparities in the spatial distribution of the overall level of national physical fitness and their sources

Table 1 shows the Gini coefficient and its decomposition results of regional disparities in the overall level of Chinese national physical fitness. In 2005, 2010, and 2015, the overall Gini coefficient of the development level of China’s national physical fitness showed a trend of first decline and then rising, the lowest in 2010 (0.0167), and 0.07 and 0.1 percentage points higher than that in 2005 and 2010 respectively. It means that during the inspection period, the overall regional gap in the development level of Chinese national physical fitness narrowed first and then expanded. In terms of sub-regions, the results of the Gini coefficient within the region show that the Gini coefficient of the overall level of national physical fitness development in the eastern region shows a trend of first decreasing and then increasing, which is consistent with the changing trend of the overall national level; the Gini coefficient of the central region has increased from 2005 The Gini coefficient in the western region gradually decreased from 0.0205 in 2005 to 0.0118 in 2015, showing a downward trend year by year. It shows that there is obvious heterogeneity in the development trend of regional disparities in the level of national physical fitness in the three major regions of China during the investigation period. The regional disparity in the development level of national physical fitness in the eastern region first narrowed and then expanded, the regional gap in the development of national physical fitness in the central region steadily expanded, and the regional gap in the development of national physical fitness in the western region decreased year by year. From the perspective of regional disparities, the gap in the development level of national physical fitness between the east and the west shows an evolution trend of first narrowing and then expanding, and its Gini coefficient is significantly higher than that of the east-central and central-west, which means that the overall level of national physical fitness The east of the regional gap is the largest in the west [12]. From the comparison of East-Central and Central-Western, the regional gap between East-Central in 2005 (0.0115) was significantly lower than that of Central-Western (0.0184), while the regional gap between East-Central in 2010 and 2015 was higher than the Midwest. It shows that the regional disparity growth rate of the development level of national physical fitness between the eastern and central regions is higher than that of the central and western regions. From the perspective of the source of the overall regional disparity and its contribution rate, the contribution rate of the inter-regional disparity is stable at more than 54%, and the contribution rate in 2005 and 2015 both exceeded 60%, indicating that the overall regional disparity in the development level of Chinese national physical fitness. Mainly due to the gap between regions. The second is the intra-regional gap, whose contribution rate is stable between 24.78% and 27.15%, and generally shows a downward trend over time. The contribution rate of hypervariable density was the lowest, reaching the highest in 2010, at 19.75%, and in 2015, at the lowest, at 10.53%, showing a change characteristic of first increase and then decrease.

### Regional disparities in the spatial distribution of male and female national physical fitness development levels and their sources

Table 2 shows the Gini coefficient and its decomposition results of the regional disparity in the development level of Chinese male and female national physical fitness. From the perspective of males, the regional Gini coefficient of the development level of Chinese male national physical fitness has increased from 0.0153 in 2005 to 0.0159 in 2015, showing an increasing trend year by year. From the perspective of the intra-regional Gini coefficient, the Gini coefficient of the development level of national physical fitness in the eastern and central regions shows an increasing trend year by year, which is consistent with the changing trend of the overall level of men in the country; 0.0182 in 2005 gradually decreased to 0.0105 in 2015, showing a downward trend year by year. From the comparison of the Gini coefficients of male national physical fitness development levels in the three regions, in 2005 and 2010, the Gini coefficient in the western region was the largest, followed by the eastern region and the smallest in the central region; in 2015, the Gini coefficient in the eastern region was the largest, followed by the western region and the smallest in the central region. Judging from the Gini coefficient between regions, the gap in the development level of male national physical fitness between the eastern and western regions showed an evolution trend of first decreasing and then increasing, and the gap was always higher than that of the eastern-central and central-western regions during the investigation period [13]. The Gini coefficient between the eastern and central regions showed a trend of first increasing and then decreasing, reaching a maximum value in 2010; the Gini coefficient between the central and western regions generally showed a downward trend. From the perspective of the source of the overall regional disparity and its contribution rate, the contribution rate of the regional disparity has been stable at more than 51%, indicating that the overall regional disparity in the development level of Chinese male national physical fitness is still dominated by the regional disparity. The second is the intra-regional gap, whose contribution rate is stable between 24.81% and 27.74%, and generally shows a downward trend over time. The contribution rate of hypervariable density is the lowest. During the investigation period, the highest value appeared in 2010, which was 21.67%, and the lowest value appeared in 2015, which was 13.87%.

From the perspective of women, the overall Gini coefficient of the regional gap and the Gini coefficient of the eastern region generally showed a trend of first decreasing and then increasing. From the perspective of the inter-regional Gini coefficient, the Gini coefficient between the eastern and western regions is still the largest. During the investigation period, its variation trend is similar to that of the central and western regions, showing a trend of first decreasing and then increasing. The Gini coefficient shows an increasing trend year by year. From the perspective of the source of the overall regional disparity and its contribution rate, the contribution rate of the inter-regional disparity is still the largest, accounting for more than 55% over the years, followed by the intra-regional disparity, but the contribution rate of the intra-regional disparity shows a downward trend year by year. The contribution rate of the over-editing density is the lowest, and the overall evolution trend during the investigation period showed an evolution trend of first increasing and then decreasing [14]. Compared with men, the overall regional disparity in the development level of female national physical fitness is significantly larger than that of men.

### Regional disparities in the spatial distribution of urban and rural national physical fitness development levels and their sources

Table 3 shows the Gini coefficient and its decomposition results of the regional disparity in the development level of national physical fitness in urban and rural areas in China. From the perspective of cities and towns, the overall Gini coefficient of the regions with the development level of urban national physical fitness was basically stable at around 0.0185 in 2005 and 2010 and rose sharply to 0.0238 in 2015. The Gini coefficients within the eastern and central regions increased steadily year by year, from 0.0152 and 0.0075 in 2005 to 0.0374 and 0.0109 in 2015, respectively. The western region showed a gradual decline, from 0.0210 in 2005 to 0.0109 in 2015. From the perspective of the Gini coefficient between regions, the Gini coefficient between the eastern and western regions is still the largest, and generally shows a trend of first decreasing and then increasing. The Gini coefficient between the peaks reached 0.0193 in 2005, and was basically stable at around 0.0145 in 2005 and 2010. From the perspective of the source of the overall urban regional gap and its contribution rate, the contribution of the regional gap in 2005 and 2010 dominated, accounting for 58.26% and 44.86%, respectively. In 2015, the contribution of hypervariable density dominated, reaching 46.32%. On the whole, the contribution of the inter-regional gap showed a significant downward trend, while the contribution of the intra-regional and hypervariable density showed a steady increase, and the growth rate of the contribution of the super-density density was significantly greater than that of the intra-regional gap. From the perspective of the development level of rural national physical fitness, the overall Gini coefficient of regional disparity and the internal Gini coefficient of the eastern region showed a trend of first decline and then increase, the central region showed a slight upward trend, and the western region showed a significant decline year by year. And by 2015, the internal disparity in the eastern region surpassed that in the western region [15]. It means that the evolution trend of the regional disparity in the development level of national physical fitness in the eastern, central and western regions is first narrowing and then widening, slightly expanding year by year, and greatly expanding year by year. From the perspective of regional gaps, the regional gap between the east-west region is still the largest, and with the passage of time, the regional gaps between the east-west and east-central regions first increased and then narrowed, and the middle- Regional differences between the western regions are just the opposite. By 2015, the regional gap between the eastern and central regions surpassed that of the central and western regions, ranking second.

### The dynamic evolution of the spatial distribution of the development level of Chinese national physical fitness

From the perspective of relative disparity, the above analysis analyzes the degree of spatial disequilibrium in the development level of Chinese national physical fitness, its composition, and sources. In the following, we further analyze the dynamic evolution trend of the spatial distribution of Chinese national physical fitness development level from the perspective of absolute disparity and spatial distribution form using a *Kernel*-density estimation method.

### *Kernel* estimation of the dynamic evolution of the overall national physical fitness level

**Fig 2:**
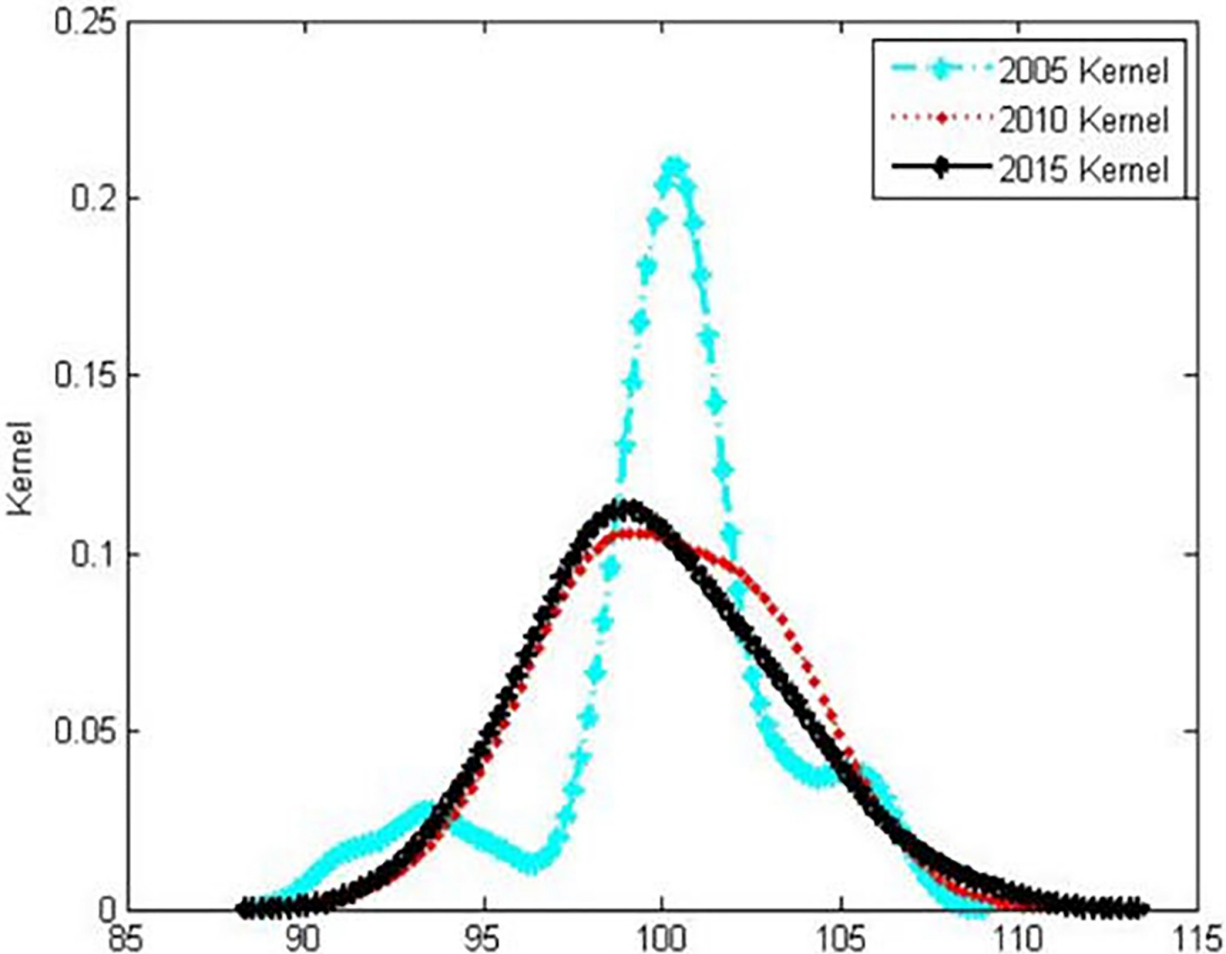
Kernel density estimation diagram for the dynamic evolution of the overall national physical fitness level.

Fig 2 shows the distribution of the nuclear density curve of the spatial distribution of the overall national physical fitness development level in China from 2005 to 2015. From this, it can be seen that the distribution curve of nuclear density in 2005 presents a two-tailed multi-peak shape, indicating that the development level of national physical fitness in some provinces is concentrated to a higher level, and the development level of national physical fitness in some provinces is concentrated to a lower level, showing a "high". The higher the higher, the lower the lower”. By 2010 and 2015, the distribution curve changed significantly: the height of the peak decreased significantly, the width of the peak increased, and the right-wing further extended slightly, showing the characteristics of evolution from a "spiky, multi-peak" shape to a "broad peak, single-peak" shape. It shows that while the overall development level of Chinese national physical fitness has improved slightly during the inspection period, regional disparities have also further widened.

**Fig 3:**
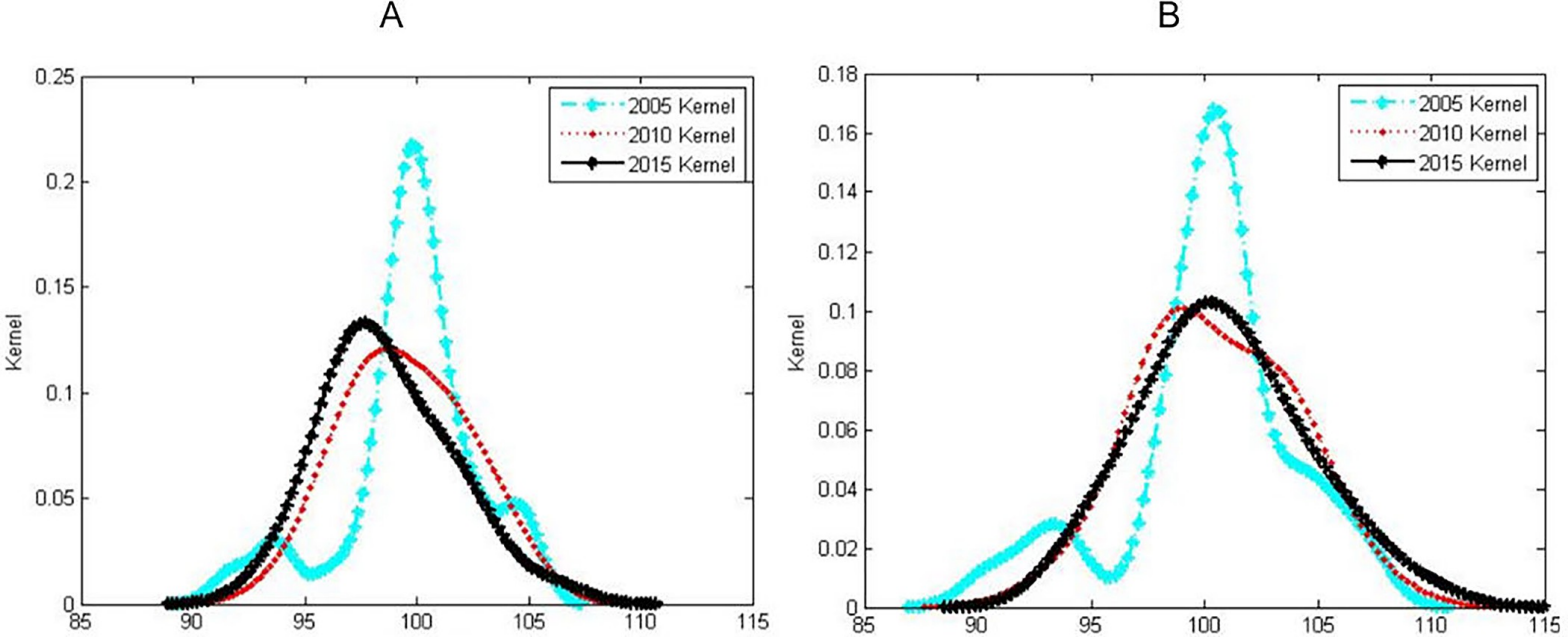
Kernel density estimation diagram for the dynamic evolution of national physical fitness level by gender (A for male, B for female).

Fig 3 is the distribution of the kernel density estimation curves of the development level of Chinese men’s and women’s national physical fitness from 2005 to 2015, respectively. It can be seen from this that the dynamic evolution trend of the spatial distribution of male and female national physical fitness development levels has a strong similarity. From 2005 to 2010, the nuclear density estimation curves of the national physical fitness development level of both males and females showed that the peak value of the peak continued to decrease, the right tail slightly extended, the kurtosis gradually widened, and the evolution from a multi-peak shape to a single-peak shape was characterized. However, the nuclear density curve did not change much from 2010 to 2015 and remained basically stable. It shows that the regional disparity in the development level of male and female national physical fitness has further increased on the whole. Among them, because the provinces with low levels of national physical fitness of men and women show a certain "catch-up effect", the polarization of low-level national physical fitness of different genders gradually disappears, while the provinces with higher development levels of national physical fitness. The physical level has also been further improved. From the point of view of the peak position, the distribution curve of females remains basically unchanged, while the distribution curve of males shifts slightly to the left, indicating that the average level of Chinese female national physical fitness has remained basically stable as a whole, while the average male national physical fitness has shown a slight decline. From the comparison of the peaks, the peak value of the nuclear density curve of males far exceeds the level of 0.1, while the peak value of the nuclear density curve of females basically remains at the level of about 0.1, indicating that the absolute regional gap in the development level of male national physical fitness is generally smaller than women.

**Fig 4:**
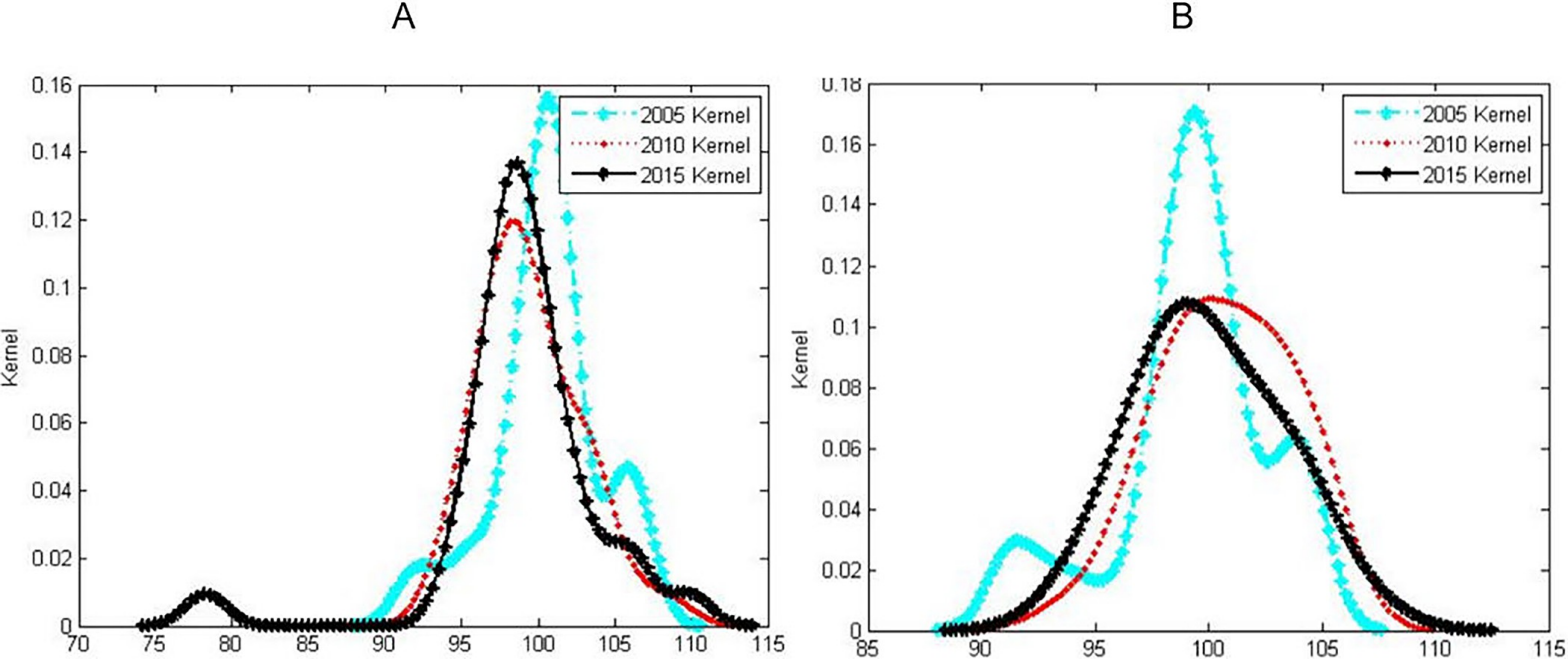
Kernel density estimation diagram for the dynamic evolution of national physical fitness level in urban and rural areas (A for urban, B for rural).

Fig 4 is the distribution of the kernel density estimation curves of the development levels in urban and rural areas of China from 2005–2015, respectively. It can be seen from the development level of national physical fitness in urban and rural areas that there is obvious heterogeneity in the dynamic evolution trend of the spatial distribution of the development level of national physical fitness in urban and rural areas in China. As far as towns are concerned, the distribution of nuclear density curves in 2010 and 2015 did not change much, but there was a big difference compared with 2005. With the passage of time, the kurtosis of the curve decreases and shifts slightly to the left, the right tail changes from an obvious single peak to a less obvious double peak, the left tail wave peak is further obvious and shifted to the left, and the left tail tip as a whole is greatly shifted to the left. Extend left. It shows that the regional disparity in the development level of national physical fitness in China’s cities and towns has generally further increased, and some provinces with relatively low national physical fitness levels are concentrated at lower levels, showing the characteristics of low-level "club convergence"; while high-level physique The trend of provincial concentration to a higher level has gradually eased. From the rural point of view, compared with 2005, the peaks of the curves in 2010 and 2015 decreased, the width of the peaks increased, and the tip of the right tail slightly extended outward, and the curves changed from multi-peak to single-peak. It shows that with the passage of time, the polarization of the development level of rural national physical fitness in China gradually disappears, and there is a "catch-up effect" in the physical development level of low-level provinces. The development trend of rural national physical fitness in different provinces tends to be Consistent.

## Discussion

Employing national physical fitness monitoring data from 31 provinces, municipalities, and districts in China for the years 2005, 2010, and 2015, this study applied the Gini coefficient, its decomposition method, and non-parametric density estimation to analyze the spatial disequilibrium and dynamic evolution of China’s national physical fitness development level. This comprehensive approach covered a diverse demographic, including males, females, urban and rural residents, providing a holistic view of national physical fitness disparities across the country.

This study introduces an innovative approach to examining regional disparities in national physical fitness. Diverging from the traditional methods of existing scholars, who typically engage in static comparative analyses using single or multiple sets of monitoring data, our research emphasizes the visibility of changing trends in national physical fitness. By meticulously dissecting the spatial differences in the development levels of national physical fitness, we employ a non-parametric density estimation system. This advanced methodological approach enables us to analyze the dynamic evolution of spatial imbalances in the development of national physical fitness.

Our comprehensive analysis not only captures the spatial and temporal dimensions of China’s national physical fitness development but also sets a robust groundwork for future investigations into the driving factors behind the spatial evolution of these fitness levels. The findings of this study are instrumental in providing a theoretical foundation for the development of region-specific policies aimed at enhancing national physical fitness across various Chinese regions. This proactive approach seeks to address regional disparities and promote a more balanced and health-conscious society.

Our analysis reveals that regional disparities in the national physical fitness development level of China’s overall, female, urban, and rural populations initially narrowed but subsequently expanded, whereas the disparities within the male population displayed a consistent year-over-year increase. This trend highlights the varying impact of national physical fitness programs and policies across different demographic segments and regions. Additionally, the study uncovers significant heterogeneity within the eastern, central, and western regions, with each region showing distinct patterns of disparity in national physical fitness levels. These findings underscore the necessity for region-specific strategies in addressing the national physical fitness needs of the population.

The results indicate that inter-regional differences are the primary contributors to the spatial imbalance in national physical fitness levels, outweighing the impact of intra-regional disparities. The east-west divide in China is particularly pronounced, suggesting a need for targeted interventions in these regions. Moreover, the dynamic evolution of the spatial distribution of national physical fitness levels across China is marked by significant heterogeneity. Over time, there has been a transition from a ’peak and multi-peak’ to a ’wide peak and single peak’ distribution, reflecting changes in regional fitness levels and disparities.

The evolution trends for both male and female national physical fitness levels are similar, yet distinct differences are observed. While the overall national physical development level has improved slightly, the absolute regional disparities have concurrently expanded. This indicates that while progress has been made in certain areas, the gap between regions with higher and lower levels of national physical fitness is widening. Provinces with lower levels of male and female national physical fitness show a ’catch-up effect,’ reducing the polarization in fitness levels across genders. In contrast, provinces with higher levels continue to improve, leading to a further expansion of regional disparities.

Firstly, in the eastern region, characterized by high urbanization and work pressure, sedentary lifestyles are common. It is imperative to motivate residents to engage in moderate physical activities. The government should amplify its efforts in health and fitness advocacy, enhancing public consciousness about the significance of maintaining an active lifestyle. Additionally, given the relatively affluent living standards in the east, where diets are often calorie-dense and rich in fats, a shift towards a balanced intake of fiber-rich foods like vegetables, fruits, and whole grains is advisable.

Secondly, addressing the western region’s notable lag in the development of national physique compared to the east, it’s crucial for the government to adopt a leadership role. This involves crafting policies that foster regional harmony in physical fitness development within a broader, holistic framework. Tailored support for the west should include graduated financial aid and optimized resource distribution, aimed at bridging the regional fitness disparity and elevating the overall standard of national physical health. Strengthening health education, promoting regular health check-ups, and encouraging the use of local natural landscapes for physical activities are also vital in enhancing the region’s health and fitness levels.

Thirdly, the central region, known for its fast-paced urban life, would benefit from a campaign encouraging increased physical activity. This could include promoting morning exercises, Tai Chi, badminton, and other fitness routines to boost physical health and wellness. The region’s diverse dietary culture should be channeled towards a balanced diet incorporating grains, vegetables, fruits, and quality proteins, while minimizing the consumption of high-fat, high-salt, and high-sugar foods.

Fourthly, acknowledging the gender-specific variations in the dynamic trends of national physical fitness across China, bespoke strategies are essential. In the eastern region, heightened oversight is needed to prevent further widening of the gender gap in physical fitness levels. Sports authorities should escalate sports promotion efforts, particularly among males, while enhancing the availability and management of public sports facilities. In the central region, additional resources and encouragement should be directed towards men in lower-index areas, advocating for a balanced diet and regular physical exercise. In the western region, targeted policies to alleviate poverty and improve economic conditions should be implemented. This includes differentiated allocation of health resources and heightened medical security, alongside gender-inclusive health education initiatives. Such measures aim to gradually level the physical development disparities between genders across regions, fostering equitable health service development for all.

In conclusion, the study’s findings highlight the need for nuanced policy-making that considers the diverse regional and demographic characteristics of China’s population. Tailored interventions are required to address the unique challenges and opportunities in improving national physical fitness across different regions and population segments. The government’s role in formulating coordinated development policies and providing adequate resources is crucial in narrowing the national physical fitness gap and enhancing the overall health quality of the Chinese nation.

## Conclusions

This study elucidates that regional variations in economic development, social welfare, and healthcare resource allocation profoundly influence the disparities in national physical health across different regions. These insights carry significant implications for the international sphere, serving as a valuable resource for countries striving to comprehend and tackle the challenges in their populations’ physical health development. The findings advocate for enhanced planning and management of medical and health resources, aiming to mitigate and prevent social inequalities. This, in turn, is pivotal in augmenting the equity and effectiveness of health services globally, thereby contributing to the promotion of social harmony and sustainable development.

Moreover, the study’s outcomes suggest a novel path forward in the realm of international physical health research. By advocating for the balanced development of national physical fitness levels worldwide, this research enriches the body of knowledge in global public health and expands the theoretical understanding of physical health. It underscores the importance of holistic approaches that consider economic, social, and healthcare factors in addressing physical health disparities. In doing so, this study not only contributes to the academic discourse but also offers practical insights for policymakers and health practitioners globally, emphasizing the need for comprehensive strategies to enhance physical health and well-being on a global scale.

## Supporting information

S1 Checklist *PLOS ONE* clinical studies checklist. (DOCX)

S1 Data (XLSX)
